# Fourteen years follow-up of a stable unilateral Keratoconus: unique case report of clinical, tomographical and biomechanical stability

**DOI:** 10.1186/s12886-022-02412-z

**Published:** 2022-06-03

**Authors:** Alain Saad, Maria Rizk, Damien Gatinel

**Affiliations:** 1 Department of Ophthalmology, Rothschild Foundation Hospital, 25, Rue Manin, 75019 Paris, France; 2 Department of Ophthalmology, American University of Beirut, Beirut, Lebanon

**Keywords:** Keratoconus, Forme Fruste Keratoconus, Corneal Tomography, Corneal Biomechanics

## Abstract

**Background:**

Keratoconus (KC) is a noninflammatory corneal ectatic disorder. In 2015, the Global Consensus on Keratoconus and Ectatic Diseases agreed that the pathophysiology of KC includes environmental, biomechanical, genetic, and biochemical disorders on one hand, and that true unilateral KC does not exist on the other hand. However, with the increasingly advancements in detection methods, we report the first case of a stable unilateral keratoconus with the longest follow up period of 14 years (2006–2020). We used topographic, tomographic, and biomechanical values for both eyes over the years to confirm the diagnosis, which has never been done before. Our study focuses on a single patient therefore it illustrates the mere possibility that unilateral keratoconus exists.

**Case presentation:**

We present the case of a 19-year-old male with no previous ocular or general health conditions who presented to our clinic in November 2006 for incidental finding of decreased vision of the right eye (OD) on a routine examination. Topographies, tomographies, and biomechanical analysis of both eyes were obtained and showed a unilateral right keratoconus at the time. Patient admitted to unilateral right eye rubbing. Although we cannot prove that previous eye rubbing alone led to these initial symptoms, he was advised to stop rubbing and was followed up without any intervention for fourteen years during which topographic, tomographic, and biomechanical values for both eyes remained stable, proving for the first time that unilateral KC could exist.

**Conclusion:**

We think that the data we are presenting is important because acknowledging that true unilateral keratoconus exists questions the genetic or primary biomechanical etiology of keratoconus versus the secondary biomechanical etiologies like eye rubbing. Our report also shows the importance of corneal biomechanics in detecting early changes. This is important to detect early, prevent progression, and tailor treatment.

## Background

Keratoconus (KC) is a noninflammatory corneal ectatic disorder, characterized by steepening of the cornea associated with progressive stromal thinning and loss of best spectacle-corrected visual acuity. It is a relatively rare disorder and its prevalence varies from 0.002% to 0.3% depending on the studied population. In 2015, the Global Consensus on Keratoconus and Ectatic Diseases agreed that the pathophysiology of KC includes environmental, biomechanical, genetic, and biochemical disorders [1]. In 2016, Gatinel hypothesized that the biomechanical changes seen in KC cannot occur without chronic eye rubbing [2]. On the other hand, the global consensus also agreed that true unilateral KC does not exist [1]. In fact, KC is known to be a bilateral disease. However, even with the increasingly advanced detection methods, there are reports of true unilateral KC with a frequency ranging from 0.5% to 4%. In this case report, we report a case of stable unilateral keratoconus that has been followed for 14 years (2006–2020). Unilateral keratoconus has been described previously [3,4], but in our case, we incorporate topographical, tomographical, and biomechanical values on one hand, and long term follow up on the other hand, making this case report unique. The fellow eye in this patient had no identifiable clinical, tomographic and biomechanical abnormalities and remains normal and stable.

### Case presentation

**Fig. 1:**
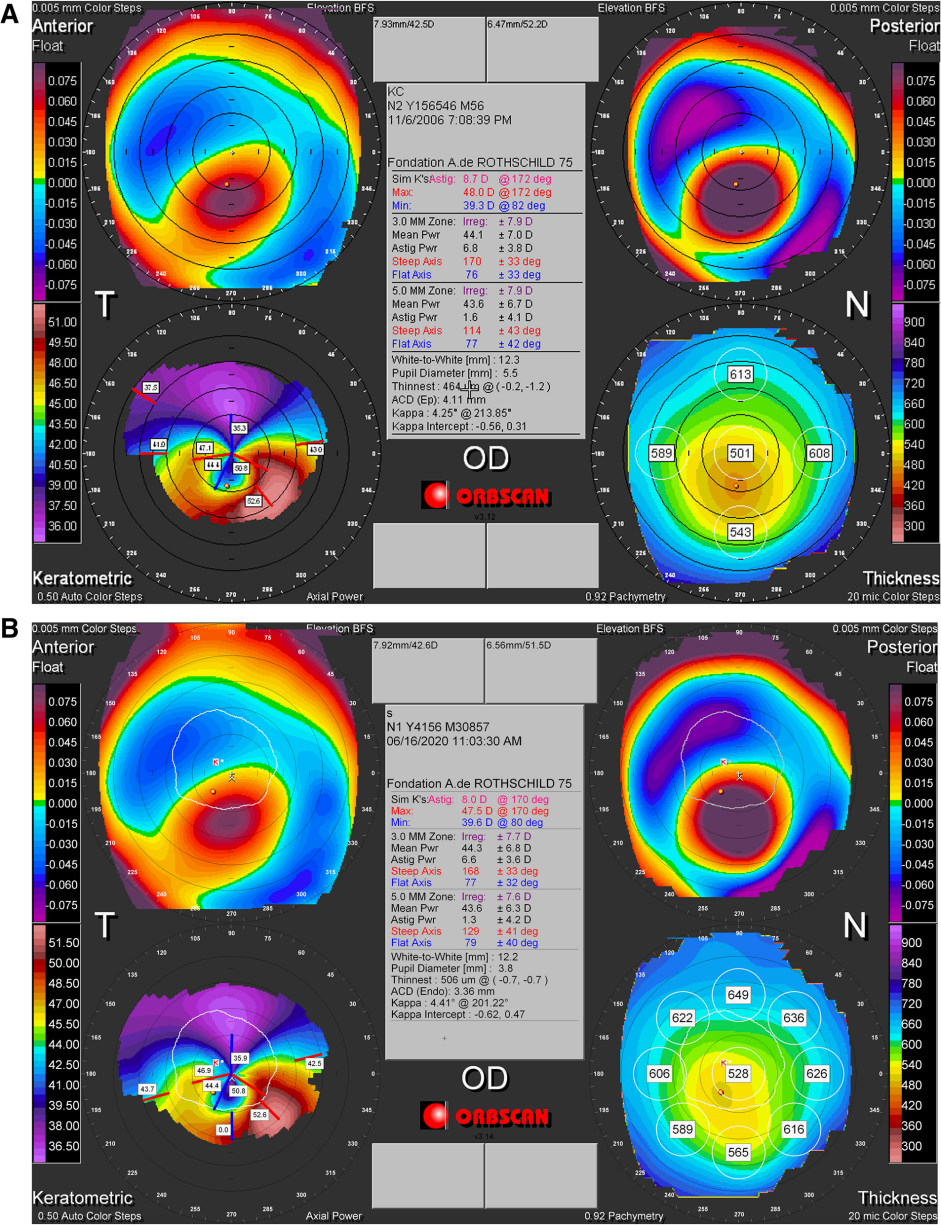
**A, B** Topography (Orbscan) OD in 2006 (**A**) remaining unchanged in 2020 (**B**) showing keratoconic changes with an inferior steepening and thinning

**Fig. 2:**
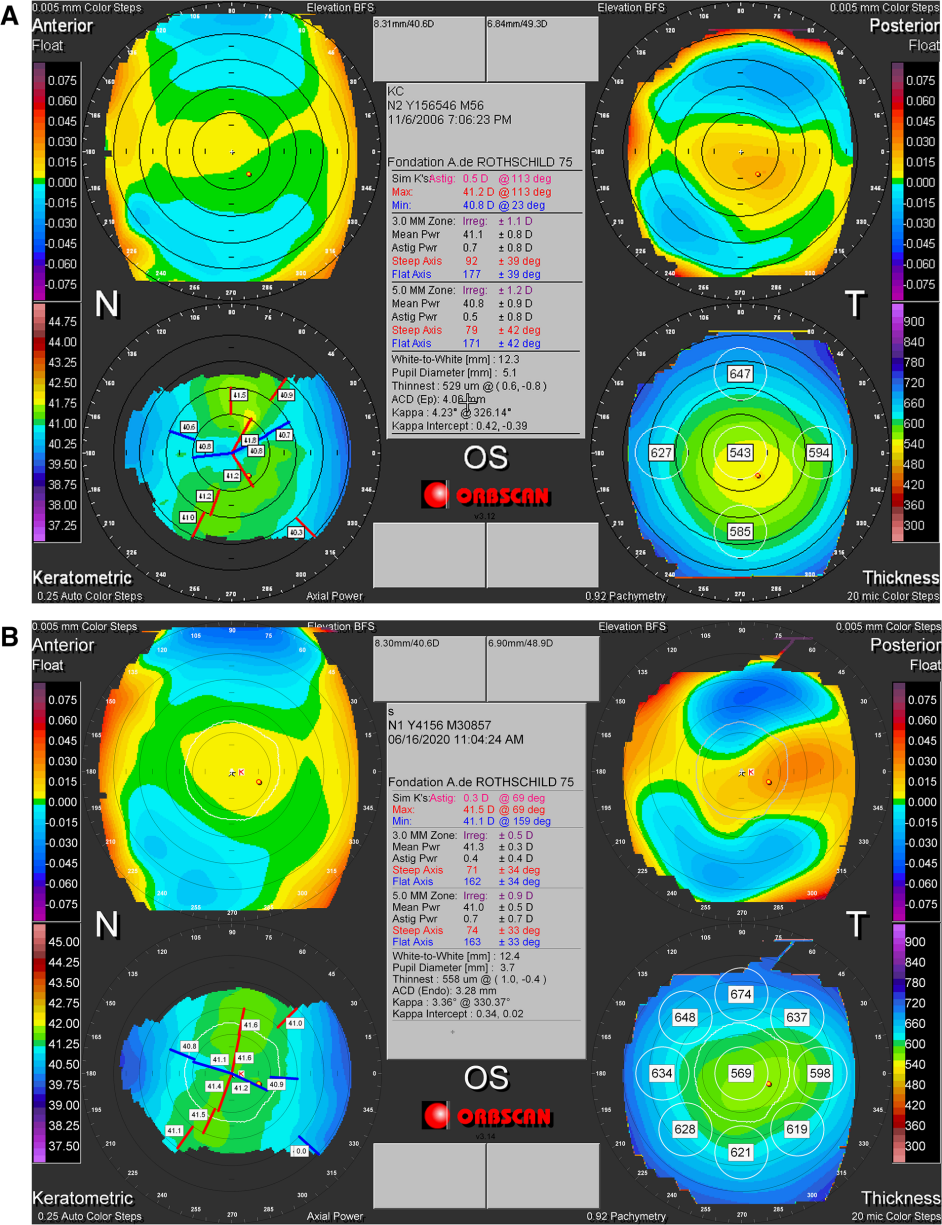
**A, B** Topography (Orbscan) OS in 2006 (**A**) remaining unchanged in 2020 (**B**) and showing within the normal topographic measurements

**Fig. 3:**
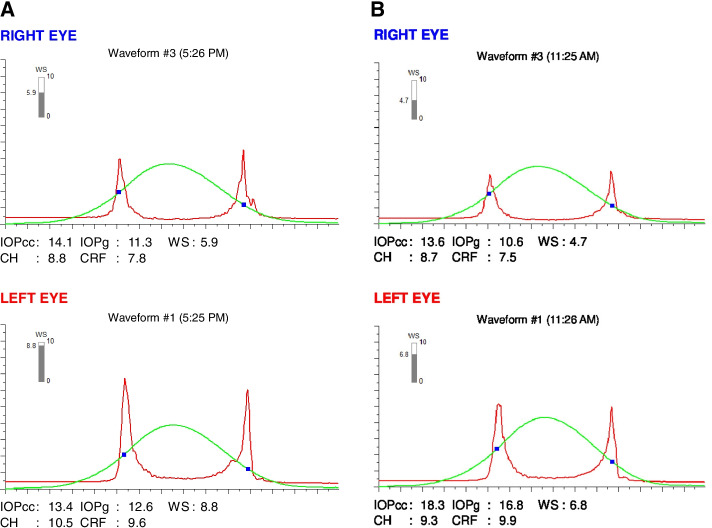
**A, B** Ocular Response Analyzer (ORA) in 2014 (**A**) and in 2020 (**B**) showing stable biomechanical values over the years OU

A 19-year-old previously healthy male presented to our clinic in November 2006 for incidental finding of decreased vision of the right eye (OD) on a routine examination. He had no prior ophthalmic or familial history. At the first visit, his best corrected visual acuity (BCVA) was 0.22 logMAR (-6.50 + 7.50 × 0) OD and 0 logMAR (-1.25 + 0.25 × 135) in the left eye (OS). The intraocular pressure was 14 mm Hg in both eyes (OU). Slit-lamp examination and fundoscopy were within normal limits OU. Corneal tomography (Orbscan, Baush and Lomb) OD showed a crab claw like appearance inferiorly with inferior decentration of the thinnest corneal point (456 μm) and a Kmax of 48.4 D (Fig. 1-A). Corneal tomography OS, however, showed a perfectly regular cornea with a thinnest pachymetry of 531 μm, a centered thinnest point and a Kmax of 41.3 D (Fig. 2-A). Patient admitted to unilateral right eye rubbing. He was counseled to avoid sleeping with pressure on his eyes and to strictly stop eye rubbing. Biomechanical analysis using Ocular Response Analyzer (ORA, Reichert) confirmed the presumptive diagnosis, showing keratoconic changes in the right eye and normal findings in the left eye (Fig. 3-A).

**Fig. 4:**
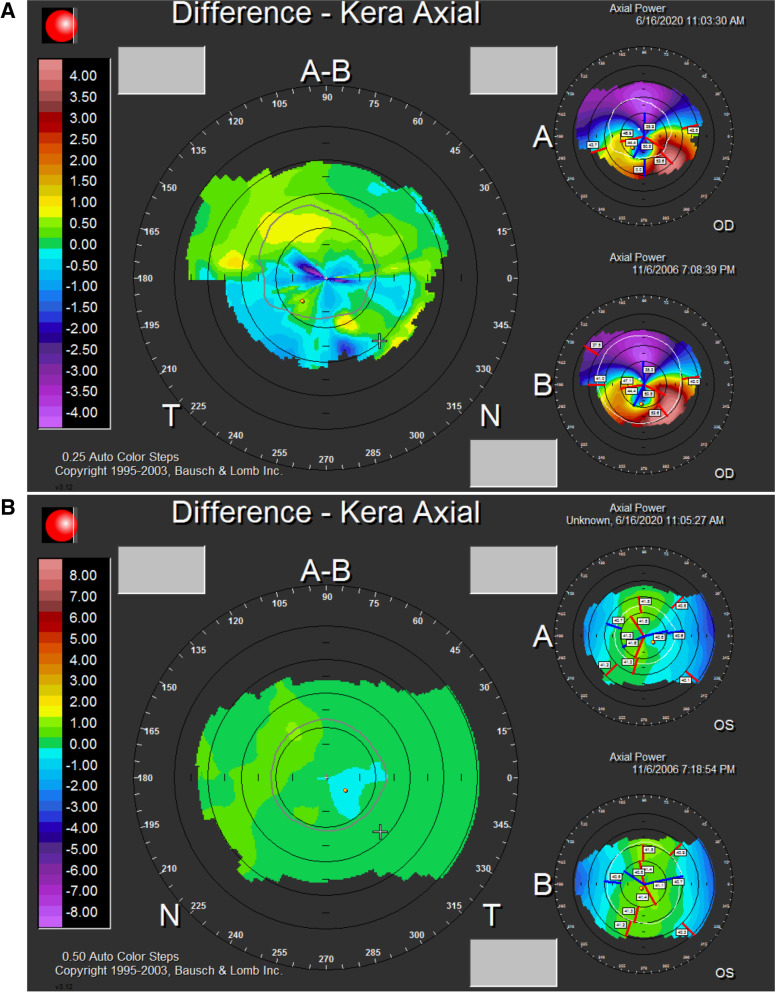
**A, B** Differential maps OD (**A**) and OS (**B**) between 2006 and 2020

**Fig. 5:**
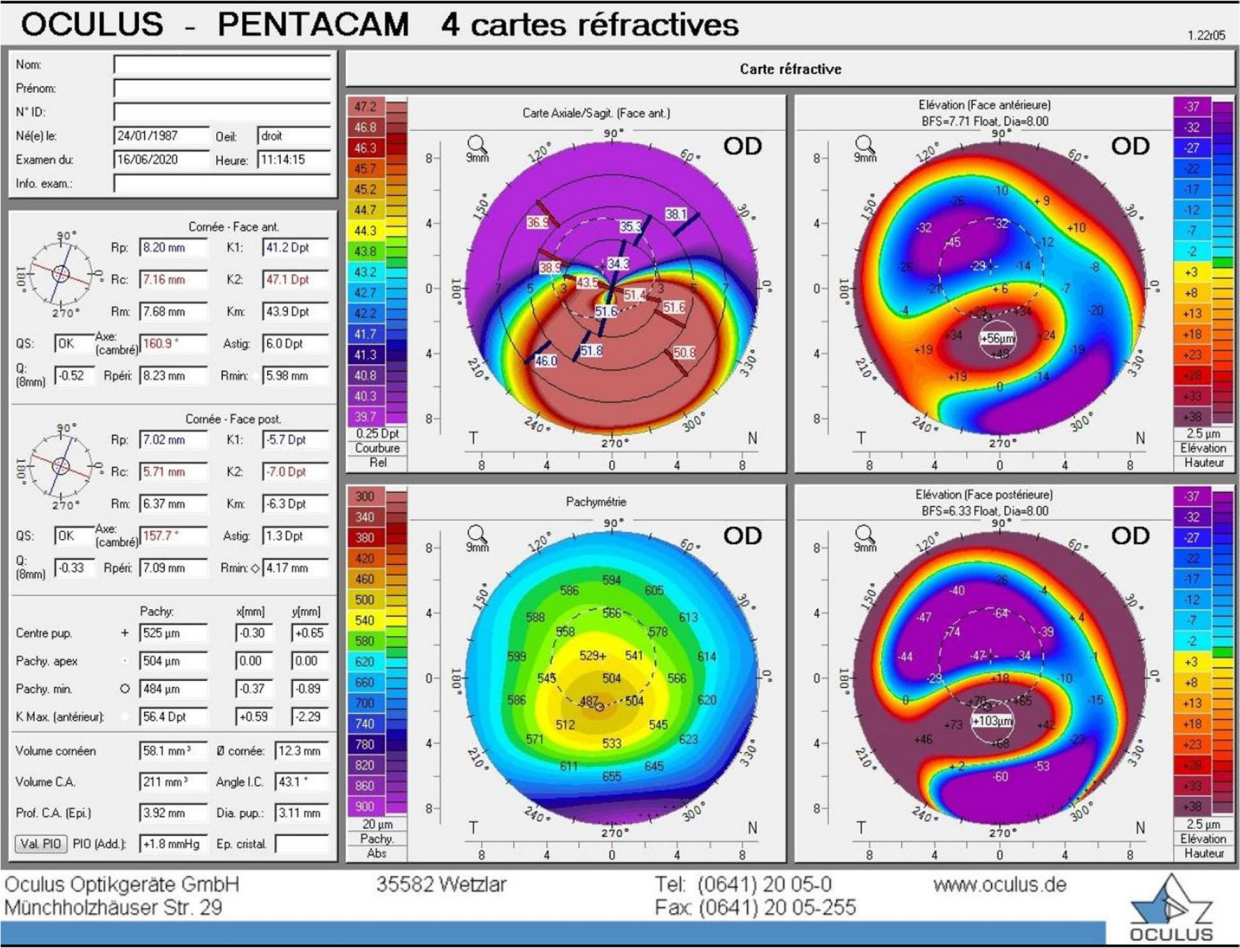
Pentacam of the right eye in 2020 showing an inferior cone with steep K values typical of keratoconus

**Fig. 6:**
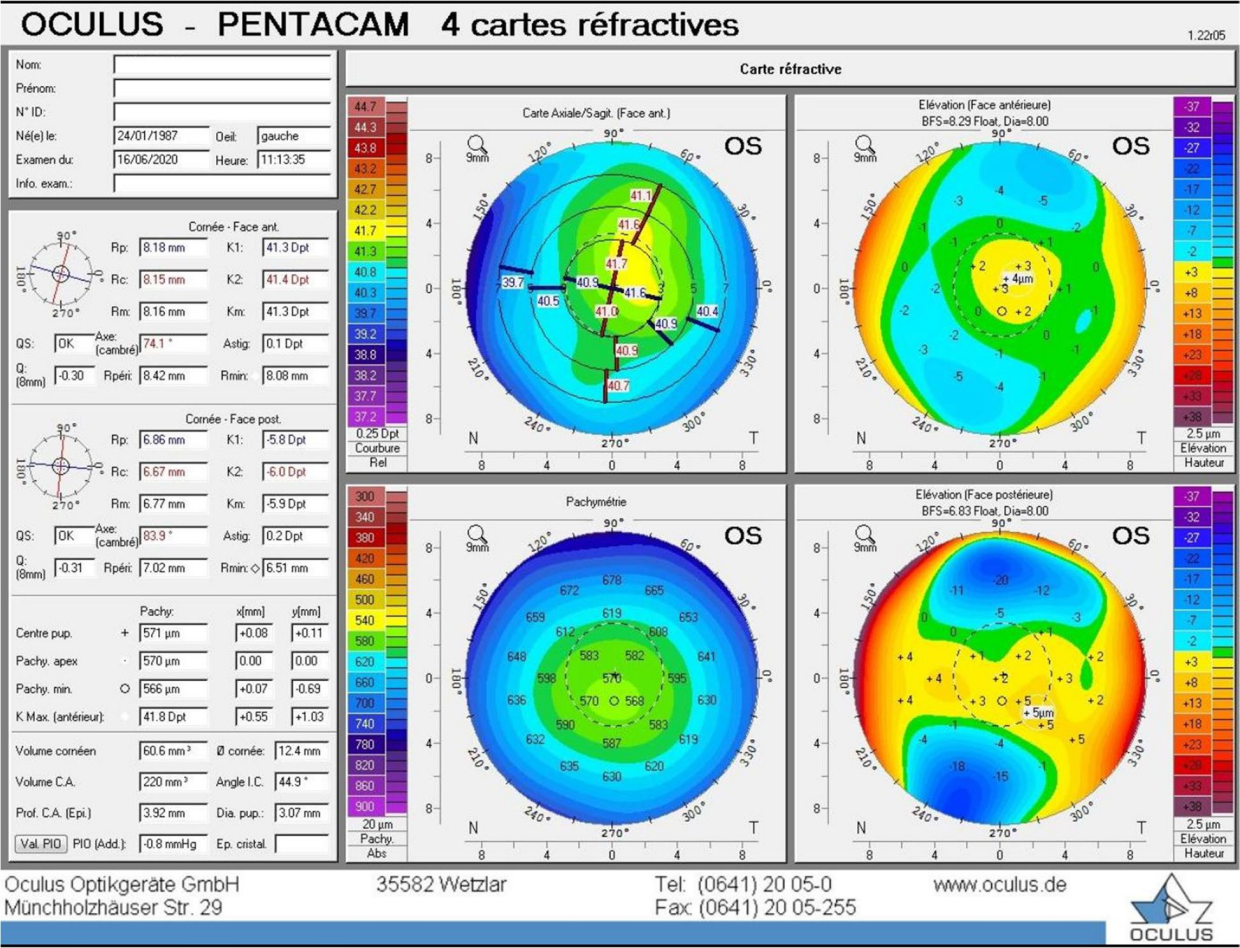
Pentacam of the left eye in 2020 showing normal anterior and posterior elevations

**Fig. 7:**
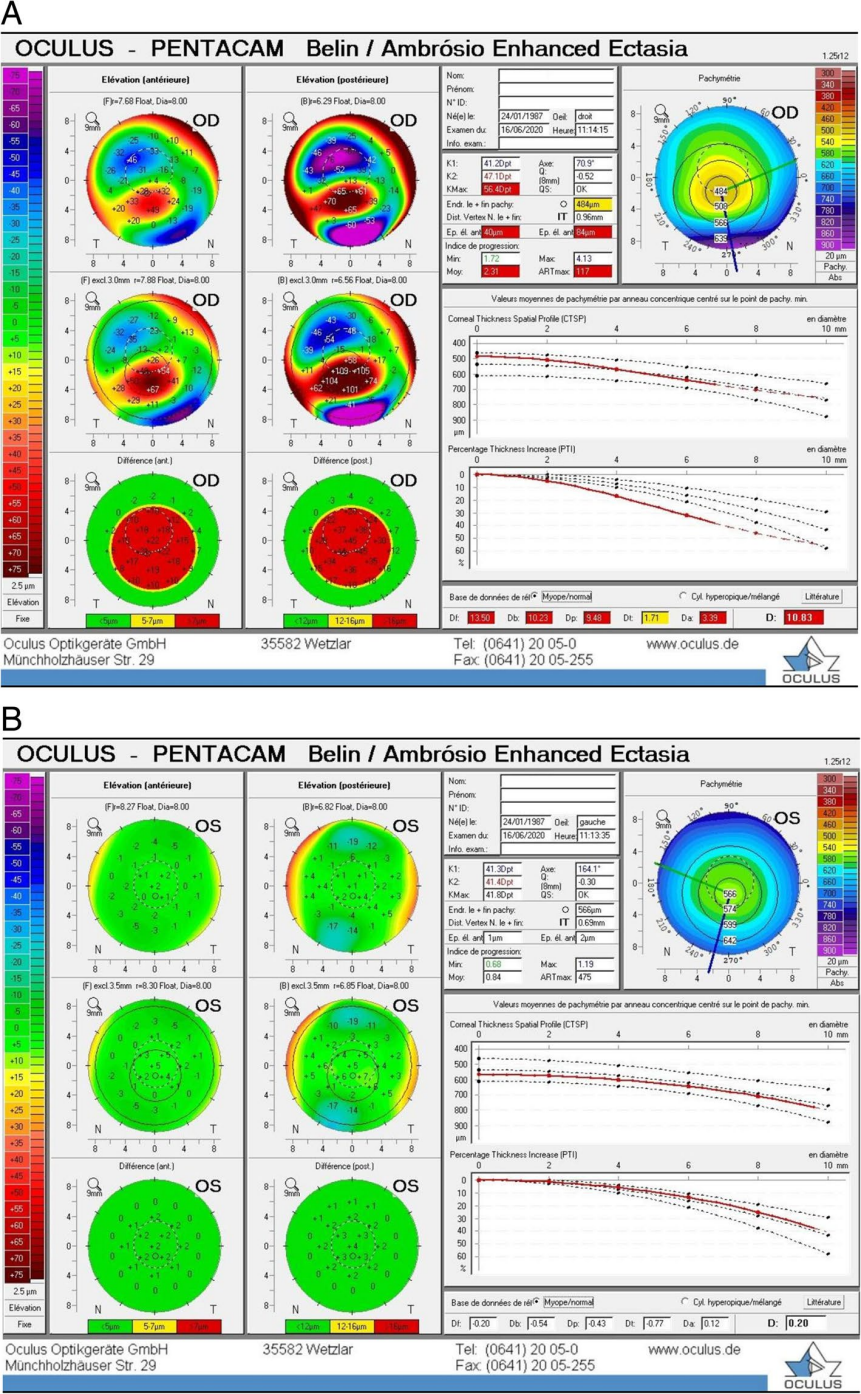
**A, B** Belin-Ambrosio enhanced ectasia report of both eyes showing keratoconus changes OD and normal anterior and posterior float values OS in 2020

**Fig. 8:**
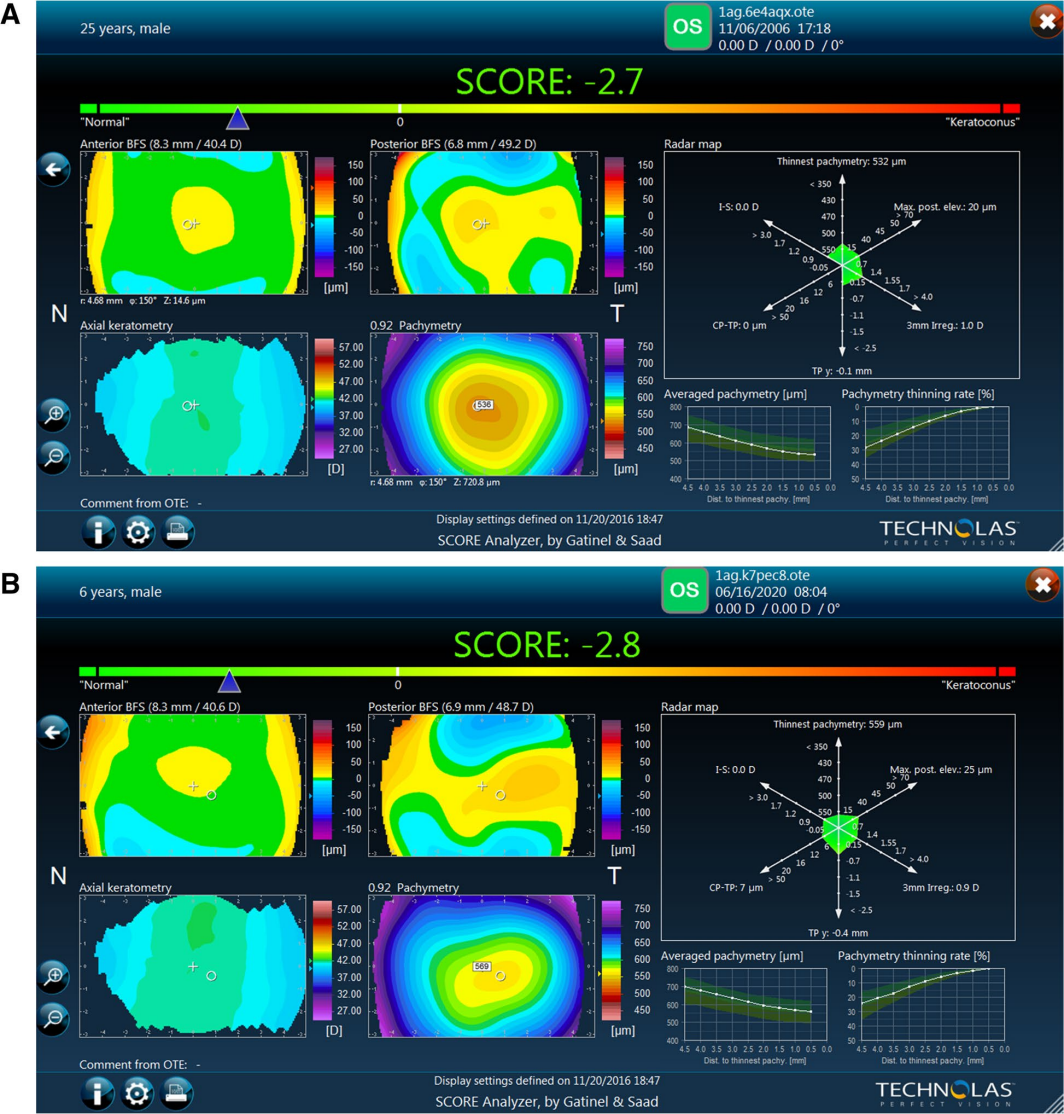
**A, B** SCORE Analyzer System in 2006 (**A**) and 2020 (**B**) showing no significant difference over time

The patient was diagnosed with a unilateral right keratoconus and was given eyeglasses to improve his right eye visual acuity as a first step. Artificial tears and anti-allergic eyedrops (Ketotifen 0.25 mg/ml) were also prescribed as permanent treatment. No further intervention was made at the time and the patient came back to clinic for annual follow ups over the years. During the 14 years of follow up we had with this patient, his refraction and visual acuity, as well as his topographies (Fig. 4, A-B), tomographies (last Pentacam images OU showing keratoconic changes OD and normal anterior and posterior elevations OS in Figs. 5 and 6) and biomechanical properties remained stable OU (Figs. 1-B, 2-B, 4-B). To further add evidence on normal posterior float of the left eye over the years, we have also included pictures of the Belin-Ambrosio enhanced ectasia report of both eyes that are strictly normal in the left eye (Fig. 7 A-B). Notably, biomechanical values on ORA including corneal hysteresis (CH) and corneal resistance factor (CRF) remained stable in both the keratoconic right eye and the normal left eye (Fig. 3-B). Also, we proved stability of the left normal eye over time using the SCORE Analyzer software which showed no signs of early forme keratoconus in the left eye [5] (Fig. 8 A-B). Facing this stability over the years, no treatment was needed so far. We thus maintained our presumptive primary diagnosis of non-progressive true unilateral right keratoconus.

## Discussion

The incidence of reported unilateral keratoconus varies depending on the methods used for diagnosis. Standard teaching is that keratoconus patients eventually develop bilateral disease if the patients are observed for a long enough period of time [6]. However, despite increasingly sensitive topographic, tomographic and biomechanical diagnostic methods, not all the fellow eyes of patients with unilateral keratoconus on diagnosis have identifiable abnormalities, even after long follow ups. Our case report is unique since it incorporates several imaging modalities during the follow up period of 14 years including corneal biomechanics which has never been reported before. It is thus the longest combination of topographic, tomographic and biomechanical follow up of a normal fellow eye in a unilateral keratoconus patient. Corneal biomechanics can predict early subtle corneal changes and thus can indicate early keratoconus. They are believed to be the first manifestation of keratoconus [6,7,8]. Having stable and within normal limits corneal biomechanics in the fellow normal eye of our patient reinforces the fact that mechanical trauma to the eye, such as rubbing, can induce ectasia on its own, as previously suggested by Gatinel [2]. Previously reported unilateral keratoconus cases either only included topographies as a non-clinical diagnosis, [3,4] or follow up time that was not long enough to confirm or infirm the hypothesis of unilateral keratoconus, or most importantly did not include secondary causes of keratoconus like eye rubbing in their inclusion criteria [4]. In fact, eye rubbing is an essential factor to consider, especially when dealing with true unilateral keratoconus as demonstrated in our patient. Not only does it indicate a risk factor we can act on to prevent progression, but it also means that primary biomechanical etiology has implications for understanding the pathophysiology of keratoconus. This case raises questions concerning the consensus statement in 2015 that true unilateral keratoconus does not exist.

This is important because acknowledging that true unilateral keratoconus may exist questions the genetic or primary biomechanical etiology of keratoconus versus the secondary biomechanical etiologies. The importance of corneal biomechanics plays a role in detecting early changes. This can have implications on understanding the pathophysiology of keratoconus to better tailor the treatment and predict the prognosis.

## Data Availability

All data generated or analyzed during this study are included in this published article [and its supplementary information files].

## Abbreviations

KC: Keratoconus
OD: Right eye
OS: Left eye
BCVA: Best corrected visual acuity
ORA: Ocular Response Analyzer
CH: Corneal hysteresis
CRF: Corneal resistance factor
OU: Both eyes
